# Telemedicine in the COVID-19 Pandemic: Motivations for Integrated, Interconnected, and Community-Based Health Delivery in Resource-Scarce Settings?

**DOI:** 10.3389/fpsyt.2020.564452

**Published:** 2020-09-11

**Authors:** Bach Xuan Tran, Men Thi Hoang, Long Hoang Vo, Huong Thi Le, Trang Ha Nguyen, Giang Thu Vu, Carl A. Latkin, Cyrus S. H. Ho, Roger C. M. Ho

**Affiliations:** ^1^ Institute for Preventive Medicine and Public Health, Hanoi Medical University, Hanoi, Vietnam; ^2^ Bloomberg School of Public Health, Johns Hopkins University, Baltimore, MD, United States; ^3^ Institute for Global Health Innovations, Duy Tan University, Da Nang, Vietnam; ^4^ Faculty of Medicine, Duy Tan University, Da Nang, Vietnam; ^5^ Center of Excellence in Evidence-based Medicine, Nguyen Tat Thanh University, Ho Chi Minh City, Vietnam; ^6^ Department of Psychological Medicine, National University Hospital, Singapore, Singapore; ^7^ Department of Psychological Medicine, Yong Loo Lin School of Medicine, National University of Singapore, Singapore, Singapore; ^8^ Institute for Health Innovation and Technology (iHealthtech), National University of Singapore, Singapore, Singapore

**Keywords:** COVID-19, telemedicine, health delivery, digital technologies, low-middle income countries

The coronavirus disease (COVID-19) has crippled health systems within a matter of months and created tremendous pressure on the physical and psychological aspects of millions of people’s lives across the world (1, 2). In such exceptionally challenging times, over 200 countries and territories have been suffering from an acute shortage of medical personnel and medical equipment (1, 2). In global efforts to prevent the spread of the virus (SARS-CoV-2), practicing social distancing and isolation is considered as an effective containment measure that has been applied around the world. Healthcare providers, therefore, are required to serve patients both physically and virtually to accommodate this historic moment.

In Vietnam, thanks to prompt and vigorous actions of the government against COVID-19, including practicing aggressive social distancing, no fatality case has been recorded, and among 324 confirmed cases, 82.1% of them have recovered and been discharged from hospital (3). However, the situation with this pandemic is still unpredictable, and people having suspected COVID-19 symptoms or outpatients in need of healthcare are facing a dilemma about whether to visit the hospital in person. Under aggressive social distancing, people were asked to avoid directly going to hospitals; instead, they should contact health advisers at the hospitals *via* telephone for early direction and services referrals. The fear of exposure to COVID-19 in addition to shortage in testing capacity and required self-isolation makes patients with suspected symptoms, undiagnosed, or untreated medical conditions experienced more physical and psychological distress.

Although Vietnam’s health system has four levels of administration, in the past years, patients prefer to have their first visit to central or provincial hospitals. This preference is partly due to the perception of higher services quality and health workers’ capacity and partially because of improved local transportation and socioeconomic status (4). Besides, when the patients have a poor prognosis of recovery, local doctors are more likely to transfer them immediately to a higher level of health services. As a result, provincial and central hospitals have always been overloaded with increased risk of hospital infection in addition to long waiting times and high non-medical costs incurring to the patients.

Accelerating telemedicine may support health services delivery systems, effective and efficient telemedicine-based health diagnosis, treatments and rehabilitation have not been widely implemented in Vietnam (5–11). Telemedicine is defined as the use of medical information *via* electronic communication, including the telephone network, computer networks, the internet, and the radio broadcasting system to deliver healthcare services at a distance (12, 13). Although its first introduction in Vietnam was in 1998 (13), telemedicine has not yet fully integrated into the Vietnamese health system due to the lack of contextualized strategies, financial and human resources, legal and infrastructure, and the culture of absorbing new technologies in health (5). The unprecedented turbulence caused by COVID-19 has acted as a catalyst to transform the healthcare service delivery in Vietnam. Diagnosis and treatment processes have been tailored to ensure social distancing; the use of telemedicine, therefore, has been strongly encouraged. Joint efforts by the Ministry of Health in coordination with the Ministry of Information and Communications has created several synchronous telemedicine projects launched on 16 April 2020. The Central Lung Hospital utilized videotelephony to examine patients *via* a smartphone application. Notably, Hanoi Medical University Hospital introduced a “digital hospital” project which aimed to shape an alternative approach for both doctor-to-patient communications and doctor-to-doctor continuing professional coaching. Preliminary results of two-month pilot revealed the promising role of telemedicine to enhance quality of healthcare service in the digital era. (14). To more effective development, it is recently prominent that the Project “Remote health examination and treatment” has been approved by Ministry of Health for the 5-year period, 2020–2025 (15). In addition, setting up community hubs for pre-checking patients and linking them to senior doctors at central hospitals have been practiced in various settings.

Although telemedicine is essential to reach the patients during social distancing, several barriers have been challenging its applications in resource-scare settings. As a country with 54 ethnic groups, 66% living in the rural, 68% using smartphone out of 89% those having mobile phones (16), localized telemedicine services have not yet established prior to the COVID-19 pandemic, thus, resulted in limited coverage of this innovation (17, 18). For multiple years, the focus of telemedicine was on technical aspects and IT infrastructure rather than interprofessional and interpersonal components in health care delivery and medical education in the “virtual” space. In addition, insufficient legislature and legal frameworks, as well as regulations on medical procedures and protocols at the national level, are confining both hospitals and health professionals to use telemedicine systems. In the light of Vietnam’s time of social distancing, despite a very strong political will on the implementation of telemedicine (19), there has not been standardized guidance for provincial and grassroots health services delivery, leading confusion in the transition to the telemedicine-related services. From the providers’ perspective, perceived barriers included the fears of medical errors, poor patient compliance, inadequate doctor-to-patient communication when using teleconsultation services, inability to monitor treatment progression. While, as for the patients, concerns regarding unclear or inadequate privacy, confidentiality, and security regarding their records, the limited IT infrastructure in the rural areas, both health and technology literacy, and missing many benefits of direct patients-doctors interactions reduce the willingness of patients to be involved in telemedical care services.

The increasing demand for telemedicine in various resource-scarce settings requires a systematic approach, not merely the switch from face-to-face to electronic- and virtual communications. In practice, this requires restructuring the organization and mechanism for healthcare delivery toward a more integrated, interconnected, and community-based approach. Central hospitals should take the advantages of setting up satellite clinics with professional medical education, technology transfer, and quality assurance. The implementation of telemedicine thus needs to be in parallel with the layers of services management, enhancing capacity and readiness across four levels of the health system concurrently, instead of being centralized. The inter-connected system will allow sharing patient records, referring, transferring, and monitoring patients in the virtual environment.

Although telemedicine can contribute to addressing the COVID-19 pandemic, it is also critical to mobilizing local resources by involving community and family in medical care, and hygiene practice is essential during the COVID-19 pandemic. Community and family are those “gate-keepers” in the frontline who direct healthcare-seeking behaviors, prevent potential infection spread, strengthening linkages between health service providers and intersectoral taskforces, and support continuing care for at-risk groups, e.g., older adults and patients with chronic conditions.

The “social distancing” model of healthcare in the current COVID-19 pandemic provides an experience valuably not only for Vietnam but also the countries having similar resource-scarce conditions. COVID-19 can be seen as a timely motivation to accelerate telemedicine-based adoption and promote a more efficient health system.

## Author Contributions

Conceptualization: BT, MH, LV, and HL. Methodology: BT, MH, TN, and GV. Writing—original draft: BT, MH, LV, and GV. Writing—review and editing: BT, MH, LV, HL, TN, GV, CL, CH, and RH.

## Funding

Research is supported by Vingroup Innovation Foundation (VINIF) in project code VINIF.2020.COVID-19.DA03. The authors declare that this study received funding from Vingroup Innovation Foundation (VINIF). The funder was not involved in the study design, collection, analysis, interpretation of data, the writing of this article, or the decision to submit it for publication.

## Conflict of Interest

The authors declare that the research was conducted in the absence of any commercial or financial relationships that could be construed as a potential conflict of interest.
